# Intensity-dependent modulation of optically active signals in a chiral metamaterial

**DOI:** 10.1038/ncomms14602

**Published:** 2017-02-27

**Authors:** Sean P. Rodrigues, Shoufeng Lan, Lei Kang, Yonghao Cui, Patrick W. Panuski, Shengxiang Wang, Augustine M. Urbas, Wenshan Cai

**Affiliations:** 1 School of Electrical and Computer Engineering, Georgia Institute of Technology, Atlanta, Georgia 30332, USA; 2 School of Materials Science and Engineering, Georgia Institute of Technology, Atlanta, Georgia 30332, USA; 3 School of Electronic and Electrical Engineering, Wuhan Textile University, Wuhan 430073, China; 4 Air Force Research Laboratory, Wright-Patterson Air Force Base, Dayton, Ohio 45433, USA

## Abstract

Chiral media exhibit optical phenomena that provide distinctive responses from opposite circular polarizations. The disparity between these responses can be optimized by structurally engineering absorptive materials into chiral nanopatterns to form metamaterials that provide gigantic chiroptical resonances. To fully leverage the innate duality of chiral metamaterials for future optical technologies, it is essential to make such chiroptical responses tunable via external means. Here we report an optical metamaterial with tailored chiroptical effects in the nonlinear regime, which exhibits a pronounced shift in its circular dichroism spectrum under a modest level of excitation power. Strong nonlinear optical rotation is observed at key spectral locations, with an intensity-induced change of 14° in the polarization rotation from a metamaterial thickness of less than *λ*/7. The modulation of chiroptical responses by manipulation of input powers incident on chiral metamaterials offers potential for active optics such as all-optical switching and light modulation.

## Abstract (web summary)

Here Rodrigues *et al*. report an optical metamaterial with tailored chiroptical effects in the nonlinear regime, which exhibits a pronounced shift in its circular dichroism spectrum under a modest level of excitation power.

Designing nano-optic modulation systems is at the forefront of photonic research as the capacity for optical switching opens doors for applications in sensing, data transfer, quantum or photonic computing and so on. Metamaterials, nanoengineered structures composed of metallic building blocks embedded in dielectric media, demonstrate extensive potential for these nano-optical applications due to their highly sensitive plasmonic resonances and subwavelength interactions that create exotic macroscopic optical phenomena. More specifically, chiral metamaterials offer an exclusive route to modulation of these resonances when interacting with circularly polarized light. In general, chiral materials are structured media that possess a subunit whose mirror image cannot be superposed onto itself. The existence of this type of structural asymmetry gives rise to light–matter interactions that are sensitive to the handedness of the circular optical polarization. The difference in absorption between the two circular polarizations is commonly known as circular dichroism and is one of the quantitative measurements of optical activity. The other, optical rotatory dispersion, describes the rotation of a plane of polarization of a linearly polarized beam as it passes through the chiral medium, and can be calculated from the circular dichroism using Kramers–Kronig relations^1^. These same definitions that were used to characterize chirality in organic media are still used today to characterize artificially structured media with large optically active responses^2^,^3^.

To design materials with artificially engineered chirality in the optical regime we look to metamaterials. Early forms of chiral optical metamaterials were designed with single layered patterns due to their relative ease of fabrication, but the chiral responses in such planar metallic structures are usually weak and typically require oblique incidence. To obtain pronounced chiroptical responses for transmitted light at normal incidence, we must imbue the mirrored discrepancy of the two circular polarizations in the material. Thus to achieve large chiroptical responses, an enantiomer must twist with the helical pitch of the polarized wave, in terms of rotation and distance travelled in the propagation direction. By integrating materials that have absorptive resonances in the prescribed frequency domain with nanoengineered structures that match the helical features of the incident wave, metamaterials with large circularly dichroic responses can be achieved. Various chiral structures explore this twist in the propagation direction including dual-layer twisted rosettes^4^, split-rings^5^,^6^, arcs^7^,^8^, gratings^9^, helices^10^, L-shapes^11^,^12^ and arrays of twisted nanorods^13^. In addition, chiral metamaterials typically have a lattice-constant factors smaller than the operating wavelength of interest, to instigate plasmonic resonances for chiral selective absorption. The strength of these chiral metamaterials has been demonstrated in their applications as broadband circular polarizers^13^,^14^, chiral mirrors^15^, negative refractive materials^16^ and large-scale chiroptical patterns^17^. While the height of chiral metamaterial research has taught us how to tailor these optically active effects, applications of nanophotonics demand that we seek out physical means to engineer active modulation into our photonic systems. This pressing need prompts us to explore all-optical control of chiroptical responses in photonic metamaterials.

As early as the 1950s, scientists theorized the opportunities available in anisotropic media when subjected to large beam intensities^18^,^19^,^20^. Higher input powers provided a change in the amount of optical rotation achieved by the anisotropic materials^20^,^21^,^22^,^23^,^24^. The researchers recognized that by introducing large electric fields into the material, it could undergo thermal self-action or fast electronic transitions, one or both of which could induce a net gyrotropic effect in the material^20^. The thermal gradient can induce a change in the refractive index of the material, whereas electronic transitions modify the bandgap of a material. The net effect of these nonlinear interactions influences the electric susceptibility, where the diagonal and non-diagonal terms of the tensor describe the manipulation of the optically induced birefringence and the nonlinear change in rotation angle respectively^19^. However it was not until 1979 that the electronic and thermal effects had been fully separated in crystals with chiroptical responses^23^. Further studies of nonlinear optical activity have been shown in liquid crystals^25^, optically active crystals^21^,^22^,^26^, chiral solutions^27^,^28^,^29^ and a single-layer anisotropic metamaterial at angled incidence^30^. Engaging the noncentrosymmetric components of the susceptibility at high input powers allows the nonlinear generated signal to surpass the linear chiroptical contrast exhibited by the chiral structure^8^,^31^,^32^,^33^,^34^.

Here we utilize the off diagonal terms of the electric permittivity tensor to manipulate the transmitted optical beam by function of input intensity. In this article, beams of varied optical power modify the two optically active signals, circular dichroism and optical rotatory dispersion, of a chiral metamaterial. The linear response of the chiral metamaterial is characterized, providing a maximum circular dichroic response of 0.58 and a maximum optical rotation of (2.3 × 10^6^)° cm^−1^. In measuring the nonlinear response, we demonstrate a blue-shift of 10 nm of the spectral resonance at 15 mW intensities and a nonlinear optical rotation coefficient of (1.76 × 10^2^)° cm W^−1^.

## Results

### Circular dichroic responses from a chiral metamaterial

**Figure 1:**
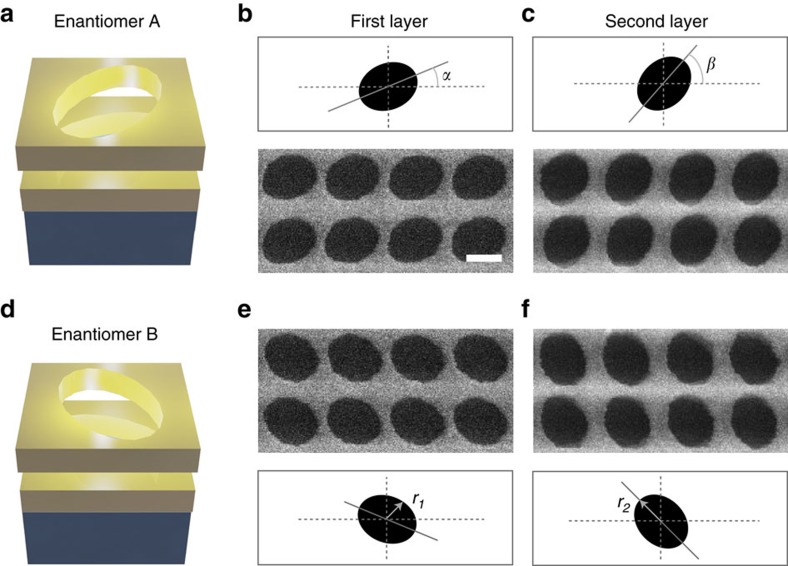
Schematic and micrographs of chiral metamaterial enantiomers. (**a**) A single unit cell of the metamaterial array. The nanoengineered material is composed of two silver films separated by 45 nm of a dielectric spacer (*n*=1.45) and perforated with two ellipses, where the major axes of the ellipses are skewed by 22.5°. (**b**) The SEM image depicts the first layer of the structure and is followed by an image of the (**c**) upper layer placed on top of the first. The scale bar in the image is 200 nm and the overall pitch of the unit cell is 350 nm. The characteristic parameters of these ellipses are *r*_1_=115 nm, *r*_2_= 150 nm and *α*=22.5°, *β*=45° from the coordinate axis. The second unit cell on the lower half of the figure is then created by aligning the ellipses such that the unit cells are mirror images of each other known as enantiomers. (**d**) Schematics and SEM images of the (**e**) lower and (**f**) upper layers of enantiomer B are provided.

The structural composition of the nanoengineered material is depicted in Fig. 1. The unit cell is composed of two 33 nm silver films that have each been perforated with an ellipse, where the two major axes are askew from each other by an angle of 22.5°. The first silver film is grown on a glass substrate and is separated from the other film by a 45 nm dielectric material with a refractive index similar to glass. The exact geometrical parameters are listed in the caption of the figure. The structure is then encapsulated within the dielectric material to prevent any form of oxidation. The encapsulation of the metallic nanostructures within the dielectric ensures that the circular dichroic response remains the same independent of the direction the sample faces when light is incident on it. A secondary unit cell is shown in the lower half of Fig. 1. This schematic is the mirror image of the upper unit cell and is known as its enantiomer. Chiral enantiomers are known to have mirror optical responses, just like they have mirror structural symmetry. An SEM image of the lower silver film is provided in the middle of Fig. 1 and a second image of the upper pattern on top of the lower film is shown on the right of Fig. 1. The orientation and size of the perforated ellipses demonstrate strong throughput in the physical structure. The alignment of the structure is well maintained, such that the lateral precision between the upper and lower patterns is within 5 nm. A similar metamaterial design has exhibited large circular dichroic responses^35^, making it an enticing structure to seek modification of its optically active signals in response to input powers.

**Figure 2:**
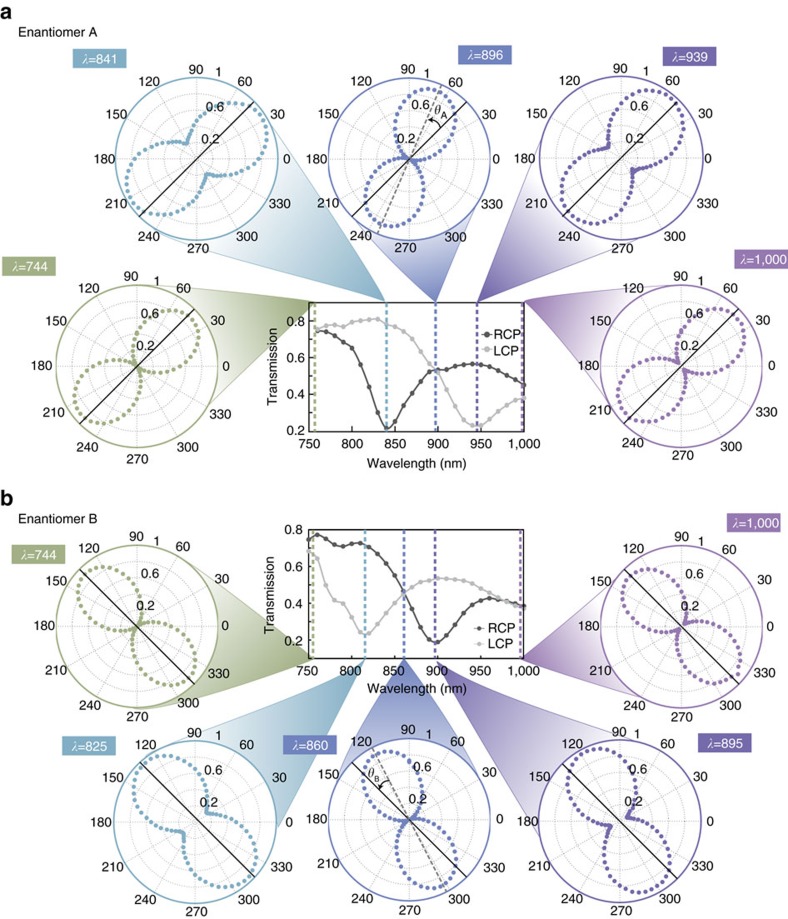
Experimental chiroptical responses for the two enantiomers. (**a**) Transmission spectra for left and right circular polarizations incident on enantiomer A. The spectra present a maximum circular dichroic response of 0.58, where CD is defined as *T*_RCP_−*T*_LCP_. Optical rotation measurements were performed with a linear polarization input at 45°, denoted by the black line in the polarization graphics. The diagrams measured at 744 nm and 1 μm show that the linear polarization and orientation is maintained as it passes through the structure. At the spectral location of the resonance dips, 841 and 939 nm, the output polarization becomes elliptical and rotated. At the spectral cross-point in the central graphic, where *T*_RCP_=*T*_LCP_ at the wavelength of 896 nm, the transmitted light remains linearly polarized, but rotated by 25° clockwise. (**b**) Mirror responses are achieved for the transmission and optical rotation data of enantiomer B, when a linear polarization is input at an angle of 135°. Similar to enantiomer A, the linear polarization is maintained at the cross-point wavelength where *T*_RCP_=*T*_LCP_, but the optical rotation is counter clockwise in this case.

To demonstrate this change in the transition from linear to nonlinear input powers, we first characterize the optically active signals of the metamaterial structure at low power exposures. Figure 2 depicts both the circular dichroic and optical rotatory responses for enantiomers A and B. The central two graphs for both enantiomers in Fig. 2a,b depict the transmission of left and right circularly polarized waves. Just as the enantiomer structures are mirror images of each other, the linear chiroptical responses as shown in the transmission graphs are also complimentary to one another. The slight differences witnessed between the two spectra can be attributed to minor fabrication deviations between the enantiomeric materials. The largest magnitude of the transmission difference between the two circular polarizations is 0.58 for enantiomer A at 840 nm and 0.50 for enantiomer B at 810 nm. This contrast is defined here as circular dichroism, CD*=T*_RCP_−*T*_LCP_. We note that each of the two enantiomer structures is designed to be optically identical from both sides and thus does not support asymmetric transmission. This circular dichroic contrast is comparable to some of the best metamaterial structures out there today^7^,^36^,^37^. The resonance dips occur over a broad spectrum in the near-infrared.

### Spectral optical polarization characterization

In addition, optical rotation measurements were performed from 740 to 1,000 nm. The five polarization figures surrounding the transmission graphics represent the state of polarization of the transmitted wave at five selected spectral regions where the chiral metamaterial demonstrates its chiral nature or lack thereof. In each polar diagram, the peanut-shaped pattern describes the measured portion of the intensity along the transmission axis of a rotating polarizer at the output. Polarization diagrams are normalized and averaged over a spectral region of 5 nm, including 30 data points to reduce noise. To assess the largest optical rotation of the transmitted light from these structures, a different input polarization angle was necessary for either enantiomer. For enantiomer A, the input polarization is 45° and for enantiomer B, the angle is 135°. This is denoted in the polarization diagrams by a solid black line at the respective angles for the two enantiomers. Intuitively, these locations are ideal for optical rotation as they are the physical locations where the two perforated ellipses intersect most closely, as seen in Fig. 1. More details on how these input polarization angles were acquired can be found in Supplementary Figs 1 and 2. As in all measurements provided in this paper, the light passes through the glass substrate first. Next, the true optical rotation should be characterized at the cross-point of the central graphic of Fig. 2. At this cross-point, left and right circular polarizations are transmitted equally, thereby allowing a linearly polarized beam to pass through without deformation of its polarization. As anticipated, the polarization diagrams for enantiomer A at 896 nm and for enantiomer B at 860 nm have a linearly polarized beam output. More importantly the linear polarization is rotated by 25° (clockwise) for enantiomer A and −20° (counter clockwise) for enantiomer B. Each of the enantiomeric metamaterials exhibit both a positive and negative cotton effect, switching optical rotation directions at both absorptive resonance as viewed in our supplementary movie. Given a thickness of 111 nm of the metamaterial structure, we realize an optical rotation of (2.3 × 10^6^)° mm^−1^ for enantiomer A and (1.8 × 10^6^)° mm^−1^ for enantiomer B. These values are among the strongest optical rotation levels in photonic metamaterials reported to date.

We can now analyse the other four polarization graphics provided in Fig. 2. The polarization diagrams that are collected at 740 and 1,000 nm show a linear polarization output at an angle that is equivalent to the input polarization. For instance, enantiomer A shows an output polarization of 45°, which is the same angle that the input polarization was incident. This congruency between the input and output polarization matches the response exhibited in the transmission spectra at this wavelength. Here both the right and left circularly polarized beams are transmitted equally, demonstrating that the structure cannot distinguish between the two input circular polarizations, and thusly has little chirally resonant-absorptive effects at these wavelengths. Two more polarization angle diagrams are provided at the resonance dips of the transmission spectra. These polarization diagrams demonstrate a loss of linear polarization, which is denoted by the increased width at the waist of the peanut-shaped intensity profile of the polarization. The manifestation of this elliptically transmitted beam located at the resonance also corresponds to the lack of transmission from one circular polarization but not the other. As a linearly polarized beam can be decomposed into both left and right circular polarizations, this loss of linearity is anticipated. This change in polarization as a function of wavelength is best understood and displayed in the attached video file published online.

### Simulated optically active responses

**Figure 3:**
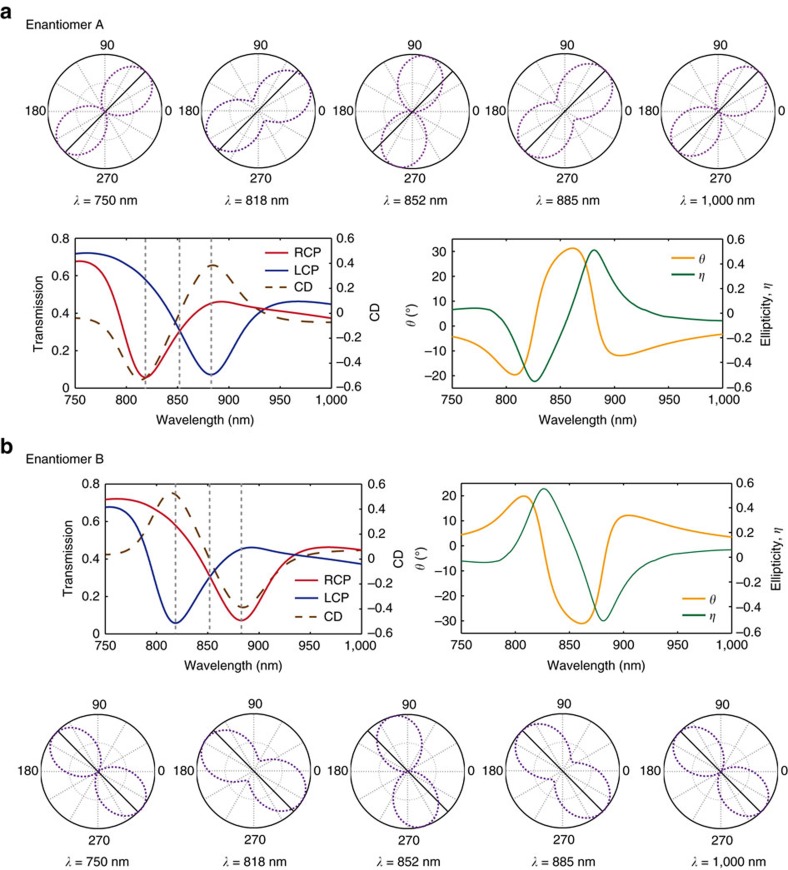
Simulations of circular dichroism and optical rotatory dispersion. (**a**,**b**) Simulated transmission spectra of circularly polarized lights on enantiomers A and B. Plots of optical rotation and ellipticity are provided. The input angle of the linearly polarized beam is the same as provided in the experimental data, 45° for enantiomer A and 135° for enantiomer B. A maximum of approximately 30° (clockwise) is obtained for the rotation of a linearly polarized beam as it passes through enantiomer A and approximately −30° (counter clockwise) as it passes through enantiomer B. The angle of rotation indicates the orientation of the major axis of the linear or elliptical beam. The ellipticity describes the shape of the polarization of the electric field, where a value of *η*=0 implies a linearly polarized beam and *η*=+1 corresponds to a right circular polarization and −1 to a left circular polarization. The responses of the optical rotation and ellipticity data are mirror symmetric for the two enantiomers.

The above-mentioned experimental results are well matched with the simulation data provided in Fig. 3. The simulated transmission spectra of circularly polarized lights on enantiomers A and B show perfectly complimentary spectra, with resonance dips at 818 and 885 nm. Figure 3 also hosts plots of optical rotation for the two materials. In the simulation, the input angle of the linearly polarized beam is the same as the one provided in the experimental data (45° for enantiomer A and 135° for enantiomer B). Maximum values of optical rotation are obtained; where the angle of rotation of the linear polarized wave as it passes through the materials is approximately 30° (clockwise) for enantiomer A and approximately −30 ° (counter clockwise) for enantiomer B. The angle of rotation indicates the orientation of the major axis of the linear or elliptical beam. In addition, simulations of ellipticity are also provided. The ellipticity describes the shape of the polarization ellipse of the electric field, where a value of *η*=+1 corresponds to a right circular polarization, *η*=−1 a left circular polarization and a value of *η*=0 implies a linearly polarized wave. The equation of ellipticity in this work is defined by the ratio of the major axis to the minor axis of the electric field or the square root of the intensity. As evident in the experimental data, the responses of the optical rotation and ellipticity data are again mirror symmetric for the two enantiomers. The graphic of the ellipticity shows a maximum and minimum, where the sign of the cotton effect changes and the direction of the optical rotation changes. As denoted by the relation of circular dichroism to the optical rotatory dispersion by the Kramers–Kronig relations, we anticipate these closely related responses.

### Intensity modulation of chiral optical responses

**Figure 4:**
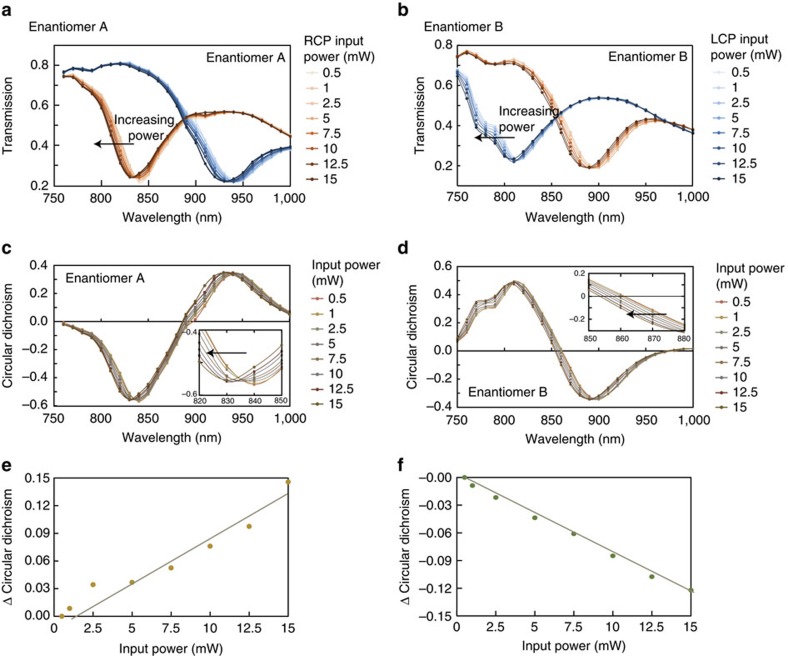
Intensity-dependent circular dichroism. (**a**,**b**) Transmission spectra of enantiomers A and B when subjected to varied intensities of right and left circularly polarized light. As incident intensities are increased, the spectra of the resonances are blue shifted. This follows that the cross points and resonance dips are also shifted, implying that the corresponding optical rotation at higher intensities will also be effected. (**c**,**d**) Circular dichroic response of enantiomers A and B as a function of input intensity. The spectral shift for the resonance is ∼10 nm for the two enantiomers as shown in the inset of **c**,**d**, where the arrows show an increase in power. (**e**,**f**) Change in circular dichroism as a function of input power for the two enantiomers. The solid line acts as a guide for the eye. A change in circular dichroism of ∼0.14 is demonstrated for a difference in power of 14.5 mW. Average excitation power of 1 mW corresponds to a peak intensity of 6 × 10^7^ W cm^−2^.

With the characterization of the linear response achieved, we can now move to study the nonlinear optical responses. Rather than investigate wave mixing signals that are generated from the chiral metamaterial as previously demonstrated^8^,^34^,^38^,^39^, we can look at the transmission property of the material as input intensities are increased. Figure 4a,b provide the transmission spectra of enantiomers A and B when subjected to varied intensities of right and left circularly polarized light. As intensities are increased from 0.5 to 15 mW a shift in the spectral resonance of the metamaterial towards shorter wavelength is observed. As the spectral location of cross-points and resonance dips are also shifted, we must later look at these new locations for nonlinear optical rotation effects. It is important to note that there is almost no threshold or definitive transition as the optical response changes from the material’s linear to nonlinear response. Rather, a gradual transition is achieved as power is increased. This leads us to ascribe this phenomenon to thermal modulation despite the fact that pulsed waves were input^24^. Figure 4c,d depicts the circular dichroic response of enantiomers A and B as a function of input intensity. The spectral shift for the resonance is ∼10 nm for the two enantiomers as shown in the inset of Fig. 4c,d. This shift occurs over a change in input power of 0.5–15 mW. Finally, Fig. 4e,f illustrates the change in circular dichroism as a function of input power for the two enantiomers. The solid line acts as a guide for the eye. The change in circular dichroism of ∼0.14 is demonstrated for a difference in power of 14.5 mW, which corresponds to approximately a 1% rate of change. Additional measurements were performed to see that the shift is not resulting from permanent damage of the metamaterial. These measurements provided in Supplementary Fig. 3, show that after input intensities were maximized to 20 mW, the power could be lowered and similar results could be achieved.

**Figure 5:**
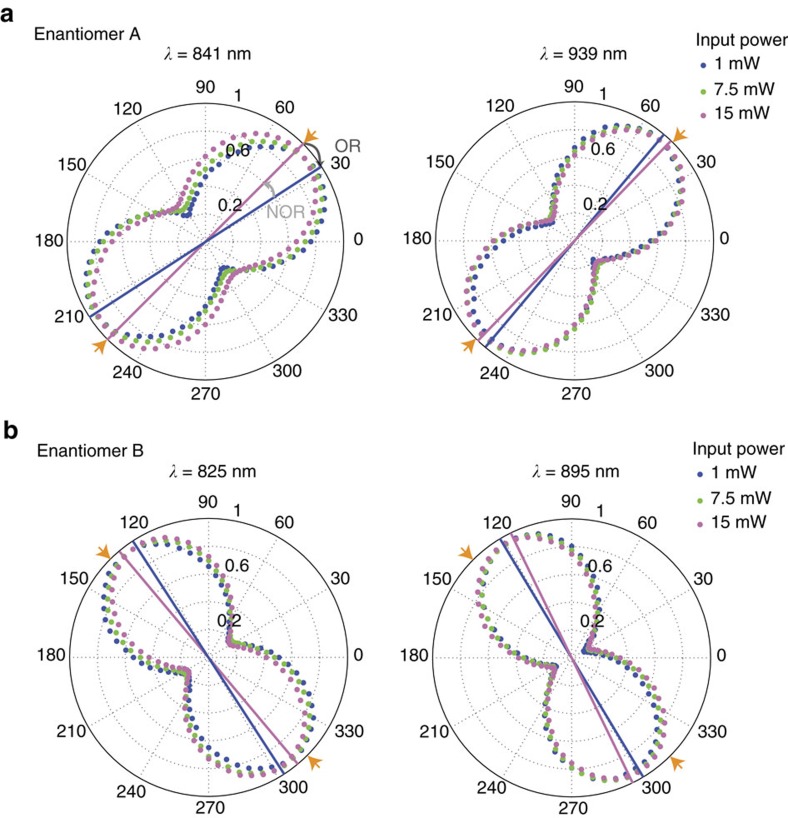
Intensity-dependent optical rotation. (**a**) Polarization diagrams of transmitted light at varied intensities from enantiomer A. Measurements of the shape of the polarization were obtained at the two resonance locations, *λ*=841 nm and *λ*=939 nm. As the power of the incident light is increased, the elliptical polarizations are rotated. The orange arrowheads indicate the input angle at which the linear polarization was incident on the sample. The blue and purple lines indicate the orientation of the long axis of the elliptical polarization at 1 and 15 mW, respectively. (**b**) Polarization diagrams of the output wave from enantiomer B. The diagrams were measured at wavelengths of 825 and 895 nm, corresponding to the resonance dips of the second enantiomer.

Nonlinear optical rotation was studied by passing linearly polarized light at varied input intensities onto the sample. The polarization of light was input at the same angles as displayed in Fig. 2, 45° for enantiomer A and 135° for enantiomer B. The nonlinear optical rotation was studied at three spectral locations—the two resonance dips and the wavelength where the two transmission curves cross, giving rise to a CD=0. Figure 5 showcases the optical rotation for three input intensities when incident on the two samples at their resonant wavelengths. First, we will discuss how the optical rotation of the outgoing polarization is affected with higher input powers. This rotation is defined by the long axis of the polarization ellipse. For enantiomer A at the first resonance, *λ*=841 nm, the polarization angle of the transmitted wave, is rotated from 31° at 1 mW to 37° for 7.5 mW and 45° at 15 mW and at the second resonance the rotation changes from 50° to 48° to 46° for the respective power increments. In this scenario, the outgoing rotation of the polarization returns towards the input polarization of 45° for enantiomer A. As for enantiomer B, the rotation of the polarization goes from 130° to 127° to 123° at the first resonant wavelength of 825 nm and 116° to 119° to 121° for the second resonance with a wavelength of 895 nm. The largest nonlinear optical rotation of the two enantiomers provides us with a nonlinear optical coefficient of (1.76 × 10^2^)° cm W^−1^. The ellipticity of the transmitted wave varies in correspondence with the input nonlinear power. As the resonance location of the metamaterial shifts with increasing beam power, the ellipticity will change in correspondence with the new resonant location at this higher input power. The changes in the waist of the peanut shape in the plots of Fig. 5 present this shift in the spectral resonance. Measurements of optical rotation were also achieved at the cross-point of the transmission curves, where the circular dichroism is equal to zero as shown in Supplementary Fig. 4. The response here is relatively weak in comparison to the response at the resonance locations. At the cross-point, the net change in transmission is zero for the two circular polarizations. As a linear polarization is a superposition of left and right circular polarizations, there is little tendency for the linearly polarized wave to rotate towards either resonance at higher powers. In the past, anisotropic measurements of nonlinear optical activity have been taken at convenient spectral locations where high laser powers are available. However, in our measurements we purposely focused on evaluating the optical rotation at the spectral locations where our metamaterial displays its strongest chiral optical activity. In doing so, we provide an in-depth nonlinear-spectral analysis of this chiral metamaterial with dual resonances.

## Discussion

We have presented a chiral metamaterial that exhibits strong chiroptical responses in both the linear and nonlinear regimes. The metamaterial bares a circular dichroic response of 0.58 and a maximum optical rotation of (2.3 × 10^6^)° cm^−1^. In the nonlinear regime, we show a spectral blue-shift of 10 nm over the entire resonance spectrum of our chiral metamaterial in response to a power change of 15 mW. This phenomenon is most likely attributed to thermal self-action of the metamaterial. To better understand these effects, a detailed theoretical analysis that is beyond the scope of this work would be desired. A change in optical rotation of (1.76 × 10^2^)° cm W^−1^ is achieved when the incident power is increased from 1 to 15 mW. To the best of our knowledge, no other material has demonstrated such a strong nonlinear chiroptical effect. Other materials in the literature and their polarization rotation per unit of optical intensity: LiIO_3_ (10^−11^)° cm W^−1^ (ref. 23); α-pinene (≤10^−11^)° cm W^−1^ (ref. 29); sucrose (2 × 10^−11^)° cm W^−1^ (refs 26, 29); an anisotropic metamaterial (3 × 10^−4^)° cm W^−1^ (ref. 30). To this extent, we believe that nonlinear optical activity can be useful in the development of optical modulation with chiral structures for applications in nanophotonic devices for optical switching and communications.

## Methods

### Sample fabrication

The fabrication of the chiral metamaterial primarily consists of a few runs of aligned electron beam lithography. The metamaterial was fabricated on a 1 × 1 inch^2^ glass substrate. Electron beam lithography (JEOL JBX-9300FS EBL) was carried out to define the lower elliptical layer. The bottom layer of the bilayered structure was formed by electron beam evaporation of 33 nm thick silver and a lift-off process. On the top of this silver structural layer, a transparent dielectric IC1-200 (Futurrex Inc.) was spin coated on. Afterwards, the dielectric was plasma etched to form a flat dielectric platform of 45 nm just above the substrate, for the subsequent patterning of the upper metallic layer. Another run of aligned electron beam lithography was repeated to form the upper elliptical array of the metamaterial. To reach the targeted performance and protect the sample from degradation, additional spin coating of the IC1-200 was performed to form a transparent top cladding of 1 μm in thickness.

### Linear optical characterization

Circularly polarized light provided by a tungsten light source modified with a linear polarizer and waveplate was incident on the metamaterial structure to measure its transmission characteristics. The measurement was performed using a homemade spectroscopy system dedicated to the characterization of small sample areas. The light was passed to a spectrometer and a silicon CCD camera. To obtain the linear optical rotation data a similar setup was utilized. A linear polarization was input on the sample and the data were then collected at an output angle every 5°.

### Nonlinear optical characterization

The excitation source for this experiment was a Ti:sapphire ultrafast oscillator (Spectra-Physics, Mai Tai HP) with a pulse duration of 100 fs, a repetition rate of 80 MHz. This laser was tuned from 740 to 1,000 nm every 10 nm to acquire the data for the nonlinear measurements above. To measure the circular dichroic response, left and right circularly polarized waves were incident on the material. The incident beam had a diameter of roughly 15.8 μm. The power impinging on the sample was controlled by a waveplate and Glann-polarizer to allow continuous modification of the optical power from 0.5 to 15 mW. The nonlinear optical rotation measurements were produced at three power levels by inputting a linear polarization at a specified input angle according to Fig. 2, and analysing the output polarization with a linear polarizer every 5°. The exact values of the maximum optical rotation were achieved by fitting cos^2^ functions to the data, where the fits achieved coefficients of determination >0.995. Average excitation power of 1 mW corresponds to a peak intensity of 6 × 10^7^ W cm^−2^.

### Data availability

The authors declare that all of the data supporting the findings of this study are available within the article and its Supplementary Information file.

## Additional information

**How to cite this article:** Rodrigues, S. P. *et al*. Intensity-dependent modulation of optically active signals in a chiral metamaterial. *Nat. Commun.* **8,** 14602 doi: 10.1038/ncomms14602 (2017).

**Publisher’s note:** Springer Nature remains neutral with regard to jurisdictional claims in published maps and institutional affiliations.

## Supplementary Material

Supplementary Information Supplementary Figures and Supplementary Notes

Supplementary Movie 1 The coordination of optical rotation and circular dichroism in a chiral metamaterial.
