# Publisher Correction: Intensity-dependent modulation of optically active signals in a chiral metamaterial

**DOI:** 10.1038/ncomms16223

**Published:** 2018-06-25

**Authors:** Sean P. Rodrigues, Shoufeng Lan, Lei Kang, Yonghao Cui, Patrick W. Panuski, Shengxiang Wang, Augustine M. Urbas, Wenshan Cai

Nature Communications 8: Article number: 14602; DOI: 10.1038/ncomms14602 (2017); Published: 02 27 2017, Updated: 06 25 2018

The original HTML version of this Article had an incorrect article number of ‘0’; it should have been ‘14602’. This has now been corrected in the HTML version of the Article. The PDF version was correct from the time of publication.

