# Supplementary material for: Intensity-dependent modulation of optically active signals in a chiral metamaterial
Source: Nat Commun. 2017 Feb 27;8:14602. doi: 10.1038/ncomms14602 (PMC5333370; doi:10.1038/ncomms14602)
Supplement: Supplementary Information — Supplementary Figures and Supplementary Notes [file ncomms14602-s1.pdf]

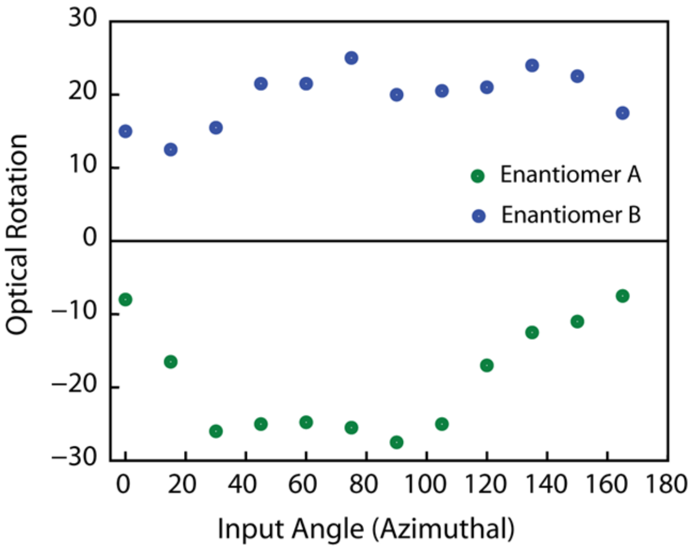

Supplementary Figure 1 | Optical rotation as a function of input angle. A linearly polarized beam is incident on the sample at 0° as denoted by Supplementary Figure 2 and rotated. The rotation of the plane wave is recorded from the transmitted light.

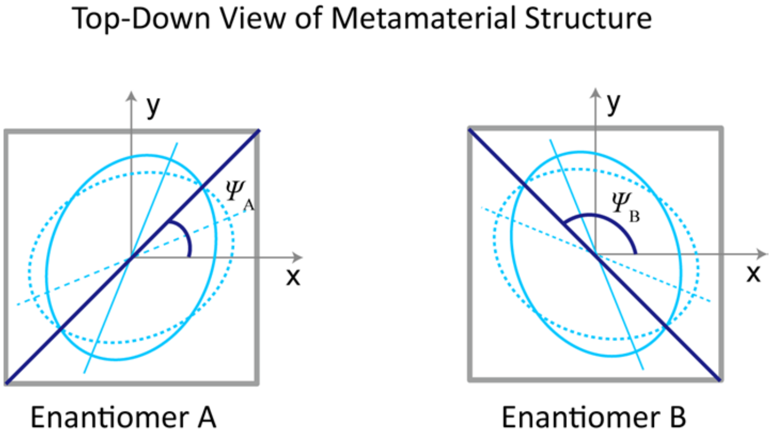

Supplementary Figure 2 | Orientation of linearly polarized wave incident on the chiral metamaterial. The dark blue line represents the input polarization chosen for the linear and nonlinear optical rotation results presented in Fig 2, Fig 3 and Fig 5. The angles Ψ_A and Ψ_B represent 45° and 135° respectively.

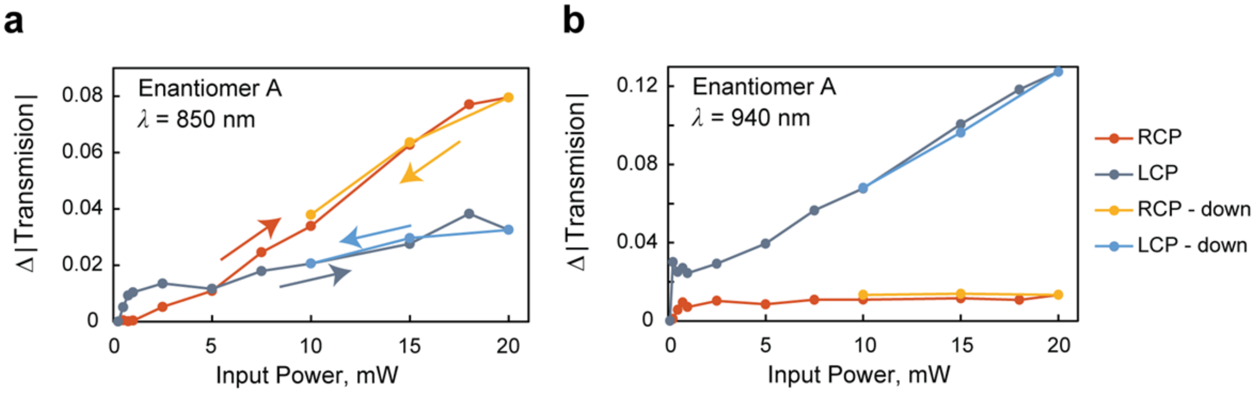

Supplementary Figure 3 | Powder dependent transmission at a single wavelength. Change in transmission as a function of input power for enantiomer A at the (**a**) first and (**b**) second resonance locations, 850 nm and 940 nm respectively.

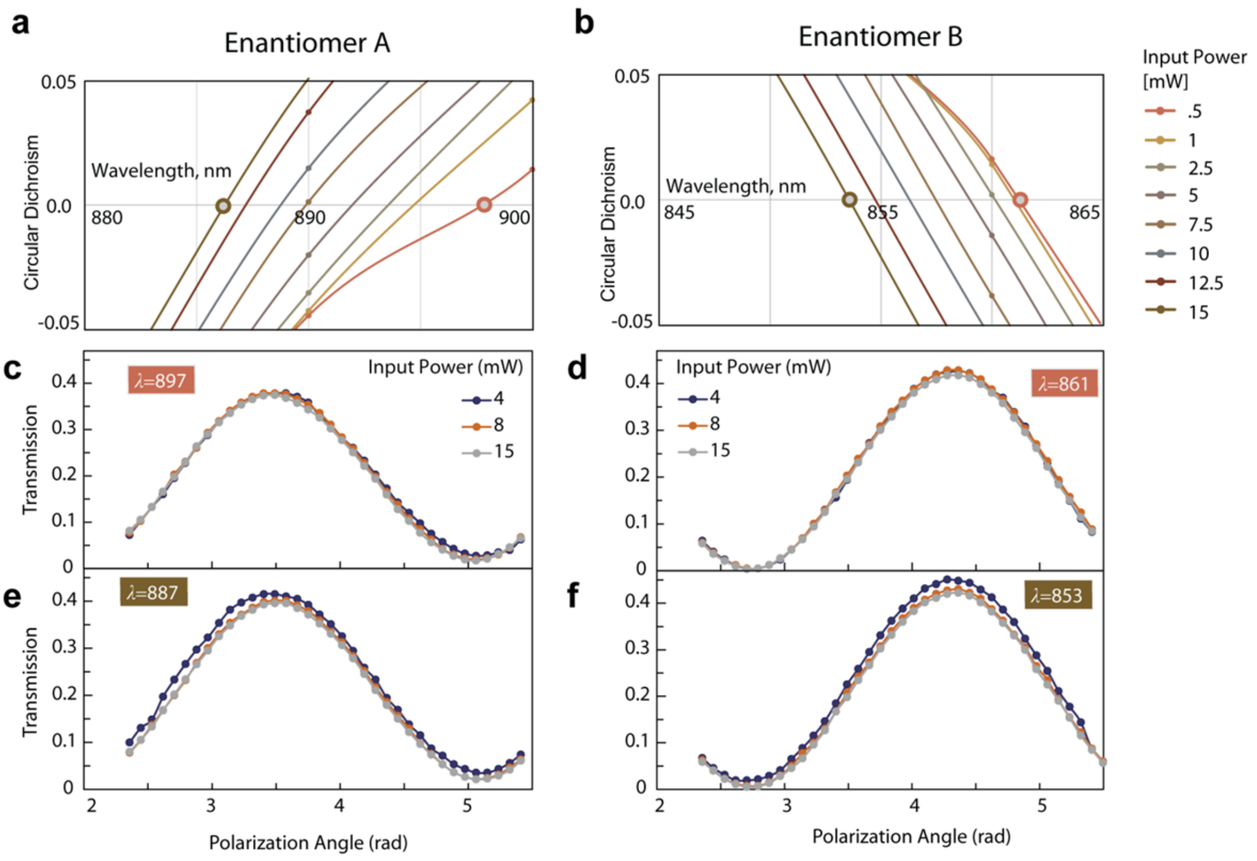

Supplementary Figure 4 | Intensity-dependent chiral optical rotation when CD=0.

(**A**) & (**B**) Spectral locations where the circular dichroic response is zero, for input intensities between 0.5 mW and 15 mW for the two enantiomers. (**C**) Intensity-dependent optical rotation at the spectral location of 897 nm for enantiomer A, where the linear response achieves a CD=0. The response shows a small polarization waist with a transmission close to zero at roughly 5 radians. (**E**) Intensity varied optical rotation at 887 nm for enantiomer B. At 15 mW, the circular dichroic response is zero at 887 nm for enantiomer A. At this spectral location, the data taken at the input intensity of 4 mW exhibits a larger polarization waist compared to that of that at 15 mW intensity. (**D**) & (**F**) Similar intensity-dependent optical rotation measurements of enantiomer B at 851 and 861 nm. The polarization waist of the 4 mW input never reaches zero, whereas the data taken at a higher intensity shows a narrower polarization waist at these blue shifted wavelengths.

Supplementary Note 1

Supplementary Figure 1 shows how the metamaterial rotates the plane of polarization when linearly polarized waves are incident at differing input angles. In order to measure this, a linearly polarized wave was incident on the sample at varied input angles and a linear polarizer was rotated to find the location of the minima. The major axis of the polarization then corresponds to ±90 of the minimized

rotation angle. It's important to note that the output polarization could vary between linear and elliptical. For this reason, when the output wave was elliptically polarized, the optical rotation corresponds to the major axis of the elliptically polarized wave. Based on these results, we chose to input the linearly polarized beam at 45° for enantiomer A and 135° for enantiomer B. The respective polarization angle is shown in correspondence to the angle of the structures in Supplementary Figure 2.

Supplementary Note 2

Supplementary Figure 3 demonstrates the change in transmission as modified by input power. As the structures are illuminated with an input power of 20 mW and subsequently tested at lower powers, it is evident that the change in transmission is not caused by permanent damage of the nanoengineered material.

Supplementary Note 3

In addition, to the nonlinear optical rotation data that was provided in the main text, measurements where the circular dichroism of the metamaterial was equal to zero were also presented. At this spectral location, the linear polarization is maintained and the orientation is rotated. However, it is important to note that this inflection point in the circular dichroic spectra shifts with varied beam powers as demonstrated in Supplementary Figure 4 (A) and (B). For enantiomer A, at low powers the circular dichroism is zero at 897 nm while at 15mw it is zero at 887 nm. For enantiomer B at low powers the CD=0 at 861 nm, while at 15 mW it is zero at a spectral location of 853 nm. For this reason, measurements of optical rotation for varied input powers were performed at both locations as seen in Supplementary Figure 4 (C)-(F). The figures show that at the blue shifted wavelengths the waist of the linear polarization at lower intensities is larger than at higher intensities as expected. A slight rotation is observed for enantiomers A and B as beam power is increased at the blue-shifted locations; this is shown by the slight degree shift in the polarization data for higher powers. In general, the nonlinear optical rotation here is relatively weak in comparison to the response at the resonant locations. At the cross point, the difference in transmission is zero for the two circular polarizations. As a linear polarization is a superposition of left and right circular polarizations, there is little tendency for the linearly polarized wave to rotate towards either resonance at higher powers.
